# The impact of the COVID-19 pandemic on child health and the provision of Care in Paediatric Emergency Departments: a qualitative study of frontline emergency care staff

**DOI:** 10.1186/s12913-021-06284-9

**Published:** 2021-03-25

**Authors:** Ciara Conlon, Thérèse McDonnell, Michael Barrett, Fergal Cummins, Conor Deasy, Conor Hensey, Eilish McAuliffe, Emma Nicholson

**Affiliations:** 1 UCD Centre for Interdisciplinary Research Education and Innovation in Health Systems, UCD School of Nursing, Midwifery & Health Systems, University College Dublin, Dublin, Ireland; 2 Children’s Health Ireland at Crumlin, Dublin; Women’s and Children’s Health, School of Medicine, University College Dublin; National Children’s Research Centre, Dublin, Ireland; 3 REDSPOT, Emergency Department, Limerick University Hospital, Limerick, Ireland; 4 Cork University Hospital, Cork, Ireland; 5 Children’s Health Ireland at Temple Street, Dublin, Ireland

**Keywords:** COVID-19, Public health restrictions, Paediatric emergency medicine, psychosocial, mental health

## Abstract

**Background:**

The COVID-19 pandemic and subsequent public health guidance to reduce the spread of the disease have wide-reaching implications for children’s health and wellbeing. Furthermore, paediatric emergency departments (EDs) have rapidly adapted provision of care in response to the pandemic. This qualitative study utilized insight from multidisciplinary frontline staff to understand 1) the changes in paediatric emergency healthcare utilization during COVID-19 2) the experiences of working within the restructured health system.

**Methods:**

Fifteen semi-structured interviews were conducted with frontline staff working in two paediatric EDs and two mixed adult and children EDs. Participants included emergency medicine clinicians (*n* = 5), nursing managerial staff (*n* = 6), social workers (*n* = 2) and nursing staff (n = 2). Thematic Analysis (TA) was applied to the data to identify key themes.

**Results:**

The pandemic and public health restrictions have had an adverse impact on children’s health and psychosocial wellbeing, compounded by difficulty in accessing primary and community services. The impact may have been more acute for children with disabilities and chronic health conditions and has raised child protection issues for vulnerable children. EDs have shown innovation and agility in the structural and operational changes they have implemented to continue to deliver care to children, however resource limitations and other challenges must be addressed to ensure high quality care delivery and protect the wellbeing of those tasked with delivering this care.

**Conclusions:**

The spread of COVID-19 and subsequent policies to address the pandemic has had wide-reaching implications for children’s health and wellbeing. The interruption to health and social care services is manifesting in myriad ways in the ED, such as a rise in psychosocial presentations. As the pandemic continues to progress, policy makers and service providers must ensure the continued provision of essential health and social services, including targeted responses for those with existing conditions.

**Supplementary Information:**

The online version contains supplementary material available at 10.1186/s12913-021-06284-9.

## Introduction

The Emergency Department acts as a gateway to the health and social care system for many children, while also providing critical care when other services are overburdened. As the COVID-19 pandemic progressed across the globe, changes in patterns of paediatric attendances at the ED indicated the pandemic was impacting children’s access to healthcare. A substantial reduction in paediatric emergency care utilization followed public health measures aimed at halting the spread of the virus, including school closures and restrictions on movement and social interactions. This has raised concerns regarding potential delays in seeking healthcare [1, 2], and increased rates of morbidity and mortality due to delayed presentations have been reported [3–5]. In Italy, paediatric presentations reduced by 73–88% compared to the previous two years [5], while in Ireland paediatric attendances fell by 46% between March and May 2020, compared to the same period in 2019 and 2018 [6]. Some studies have examined the scale of delayed presentations [7, 8], with one study finding that 32% of paediatricians working in EDs and Paediatric Assessment Units in Ireland and the UK observed delayed presentations during the initial phase of the pandemic [8]. Lower attendance rates have been attributed to reduced infectious diseases and injuries due to public health measures implemented. Furthermore, parental hesitancy to attend hospitals due to fear of COVID-19 and a misinterpretation of public health messaging may have also contributed to reduced presentations [9].

The COVID-19 pandemic has psychosocial implications for children, who may be affected in multifaceted ways on an individual, familial and community level [10], with evidence suggesting that quarantine conditions have adversely impacted children’s mental health [11]. The disruption to educational, social and recreational activities removed children from peer networks and social interaction, which may cultivate social isolation [12]. The disruption of schools and community services, often a safety net for children, may further leave children vulnerable, particularly those at heightened risk of neglect [1]. Furthermore, children with pre-existing behavioural conditions, such as autism, and those who rely on specialist or community delivered health services, may be disproportionally affected by not having their needs met [11, 13].

Health care systems have rapidly implemented protocols, redeployed staff and redistributed resources to create adequate capacity to deal with the expected surge in demand [14, 15]. As children are by and large asymptomatic and mortality rare [16], the pandemic is manifesting as a logistical, rather than clinical, challenge for PEDs [1, 14]. EDs often act as a point of entry for hospitals, therefore a robust and rapid response is crucial to ensure appropriate management of patients and infection control [2, 14]. As the pandemic progressed, it was expected to present additional challenges to healthcare systems due to the onset of the winter season, as well as schools remaining open in some jurisdictions. Moreover, in contrast to the first wave of COVID-19, health systems are trying to maintain facilitation of routine non-COVID-19 care. Documenting the response of PEDs to the pandemic can provide learning for health systems and policymakers for future healthcare planning and policy [14].

A number of studies have documented the changes to paediatric emergency healthcare, however to the best of our knowledge, no qualitative study has been undertaken to examine and contextualise these changes further. Furthermore, there is limited understanding as to how PEDs and the staff working there have adapted the provision of care during COVID-19. This qualitative study will provide rich data from the perspective of multidisciplinary frontline staff delivering care to paediatric patients in the ED in order to apprehend the already documented changes in presentations, as well as to understand the experience of working under the restructured health system in paediatric ED’s in Ireland.

## Methods

This study is part of a wider project looking at paediatric emergency healthcare utilization during COVID-19 in Ireland [17]. The main aim of this qualitative study was to utilize the perspective of ED staff to 1) understand changes to paediatric emergency healthcare utilization 2) understand the experiences of working within the restructured health system from the perspective of frontline staff. Qualitative methodology was chosen as it elicits rich data and detailed insights of the phenomenon at hand [18].

### Recruitment

Frontline emergency medicine staff working in four hospitals in total: two paediatric EDs and two mixed adult/paediatric EDs were recruited through convenience sampling. Their contact information was provided to the researcher, who in turn provided them with further information on the study and obtained written consent.

### Data Collection & Analysis

Interviews were carried out by one researcher (CC) remotely through phone or video call between August and October 2020. Eighteen potential participants were identified, three of whom were ineligible as they did not work in an ED during COVID-19. The interviews were semi-structured, conducted in line with an interview schedule developed with input from research collaborators working in clinical settings (see supplementary material). Interviews were recorded and transcribed into Microsoft Word.

Thematic Analysis (TA) was utilized, which is a widely used, flexible method and provides researchers with a systematic process to identify and analyse themes to create an analytical narrative of the data collected using NVivo™ software [19]. This began with familiarisation, followed by a recursive coding process as outlined by Braun and Clarke (2012). One researcher (CC) carried out the analysis, with 40% of interviews double coded (GO’D) to ensure consistency and methodological rigor in the interpretation of the data. Initial codes were generated, followed by clustering and collapsing codes to create theoretical themes. A reviewing process, involving discussion with all authors, was then carried out to ensure the themes represented patterned and shared meaning with relevance to the research question at hand [19].

### Ethics

Ethical approval was obtained from the COVID-19 National Research Ethics Committee (NREC) in Ireland (ref: 20-NREC-COV-034).

## Results

**Fig. 1:**
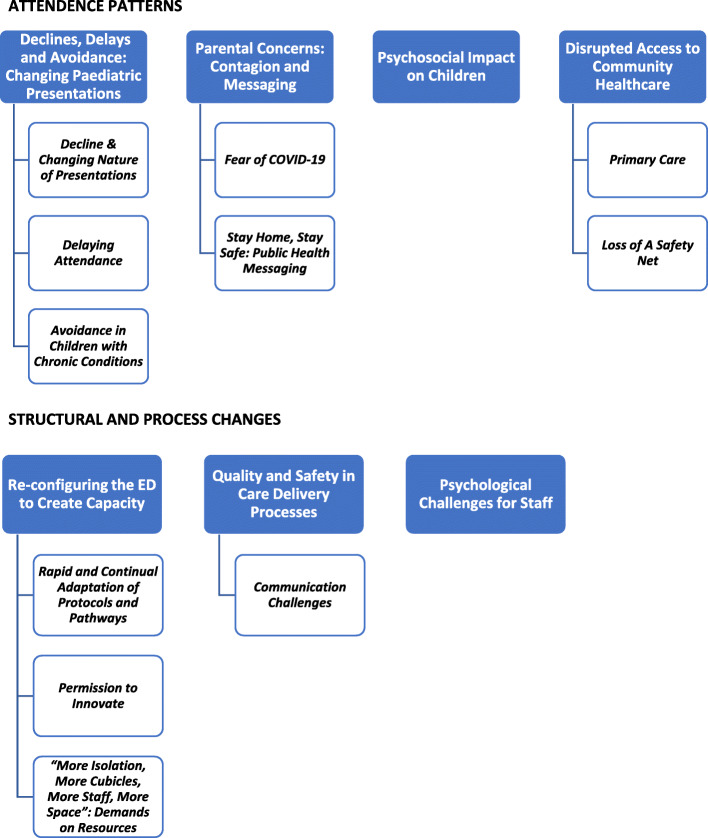
Themes & Subthemes

Participants (*N* = 15) included emergency medicine clinicians (EMC) (*n* = 5), nursing managerial staff (NMS) (*n* = 6), medical social workers (MSW) (*n* = 2) and nursing staff (NS) (n = 2). Ten participants worked in paediatric only hospitals, while five were based in joint adult/paediatric hospitals. The majority (*n* = 13) of participants were female. Seven main themes were identified, as outlined in Fig. 1.

## Attendance patterns

### Declines, delays and avoidance: changing Paediatric presentations

#### Decline & Changing Nature of presentations

Clinicians noted attendances *“absolutely dropped*” initially, however attendance began to steadily increase over the summer months. The “*bread and butter”* of paediatric presentations such as infectious diseases, viral illnesses and gastroenteritis cases notably decreased, while presentations relating to injuries and accidents, such as household injuries and accidental ingestions, decreased. The implementation of remote consultations in primary care was highlighted as changing the nature and quality of some referrals, as the absence of a physical assessment led to some patients who would typically be treated in general practice being referred to the ED, having a “*ripple*” effect on EDs:

“*we ended up seeing them in the A&E department which was something we probably wouldn’t have had to if they had done a full assessment in the GP surgery*” [Participant 1, Female MSW]

#### Delaying attendance

Delayed presentations to the ED were observed by all clinicians, particularly early in the pandemic. These delays occurred due to parental concerns and perceptions of the effect of the pandemic on the health system. Clinicians relayed incidents whereby children presented with more progressed or critical illnesses, such as diabetes ketoacidosis, malignancies, pneumonia, and meningitis: *“there was definitely a sense that we were seeing sicker children”* [P10, Male EMC]. In a small number of instances, this led to prolonged treatment or worsened outcomes for children, which clinicians stressed that while uncommon, it was a concerning phenomenon. In one instance, a child who had displayed concerning symptoms for a number of days before attending was diagnosed as having suffered a stroke, which the clinician thought could have been managed better if treated earlier:

“*that was delayed presentation as in the management of his care you know, could have been less severe had he presented earlier”* [P14, Female NS]

The child’s parent had outlined their concerns in presenting due to the risk of contracting COVID-19 and passing it to a high-risk elderly grandparent living in the same household. In another case, a clinician described a patient who had been unwell for two weeks before presenting, leading to an ICU transfer, again due to parental fears of contracting COVID-19.

#### Avoidance in children with chronic conditions

Children with complex needs and chronic conditions were identified as a cohort that were presenting less, which was understood to be due to families decision to “cocoon” at home: *“They were trying to stay away with their co-morbidities*” [P5, Female NMS]. One clinician, who works with children with disabilities in a volunteer capacity, stated families were very reluctant to allow support services into the home due to fear of infection. Concerns were expressed for families left with very little support who would normally rely on services to provide them with care and respite, particularly children requiring a high level of complex care.

*“I know everybody was housebound, but these are parents with you know, very high, having children with very high needs that they kind of weren’t receiving the supports that they probably needed*” [P13, Male EMC]

However, clinicians also highlighted alternative pathways to care implemented, such as telehealth consultations and virtual clinics, providing access to medical support which may have mitigated the need to present to the ED.

### Parental concerns: contagion and messaging

#### Fear of COVID-19

According to clinicians, parents displayed high levels of reluctance to attend the ED, predominantly driven by a fear of contracting COVID-19; as one clinician put it, they “*saw the hospital as a place of you know, risk of contracting COVID*” [P10, Male EMC]. These concerns were cited when explaining the reason for delayed presentations.

“*they felt they would have presented a bit sooner that they didn’t want to come to a department where they could have had a risk of COVID”* [P8, Female NMS]

In the beginning of the pandemic, parents rang EDs “*non-stop”* to enquire about the presence of COVID-19 in the hospital. Many clinicians sensed that parents were weighing up the risk of attending the ED against the severity of the child’s illness, and were more inclined to *“wait and see*” by managing the illness at home:

*“they were pushing their own boundaries and were allowing themselves a little bit more risk or their perception of risk before seeking medical advice because probably the perceived risk of a well child with a temperature was less than the perceived risk of coming here and being exposed to COVID-19”* [P10, Male EMC]

Parents were anxious to socially distance from other patients, and as spatial limitations often led to difficulty in implementing social distancing, parents would often wait outside the building. Clinicians explained that parents were unaware of the infection control measures put in place, such as streaming and triaging for COVID-19, but reacted positively and felt reassured when observing the measures implemented:

“*I think most of them were very reassured when they came in because they were told no, we had a separate COVID streaming area.*” [P9, Female EMC]

#### Stay home, stay safe: public health messaging

As cases of COVID-19 first began to increase in Ireland, authorities introduced restrictions on movement and issued public health advice instructing people to stay at home. The initial drop in ED attendances coincided with this messaging and a number of clinicians felt parents may have believed they should not attend hospitals, particularly for issues that parents perceived to be not absolutely urgent:

“*it was like everybody was being told do not attend your ED, do not attend your ED, do not attend your ED*” [P4, Female NMS]

*“parents were less inclined to present maybe with the normal bits and pieces because they were told to cocoon and stay at home”* [P9, Female EMC]

One clinician felt the strength of the message to “stay home, stay safe” was quite effective, and some parents were unwilling to go against this in case they put their child at risk:

“*I think because the governments message was so good initially of stay home stay safe, it really was gosh if we break that, could he really be at risk of picking up COVID and dying”* [P2, Female NMS]

Another clinician relayed an interaction with a parent who misinterpreted the government message to stay home as including being unable to travel to the hospital:

*“she fully believed that if she was stopped by the guards [police] on the road that they would tell her to turn back”* [P11, Female MSW]

Media depictions of *“front line staff being to the pin of their collar”* [P13, Male EMC], may have impacted parents decision to present as they believed hospitals to be overwhelmed with COVID-19 patients. Parents were extending apologies to staff about their attendance:

*“we didn’t want to come in, you know we didn’t want to, we knew you were busy”* [P13, Male EMC]

Parents were citing their awareness of health system capacity as a reason for delaying presenting, such as the case of one child who presented many days after suffering a serious laceration:

*“The mum was reluctant because she said she thought we’d be out the doors with sick children, so she didn’t want to be just turning up with a cut – which wasn’t really a cut at all, it was quite a serious injury”* [P15, Female NS]

Clinicians pointed to the need for a public communication strategy aimed at reassuring parents of both the infection control measures in EDs, as well as to promote the message that EDs are fully operational, which could alleviate parental concerns and prevent delayed attendance or avoidance:

*“I think if we can..[]..present to the public that we’re open..[]..if you need to come to hospital we’re ready to help you but we have these measures in place to keep it as safe as possible”* [P11, Female MSW]

### Psychosocial impact on children

Children’s mental health and wellbeing appears to have been impacted by the pandemic, which manifested in a reported rise in presentations relating to psychosocial issues, both in those with existing mental health conditions and first time presentations. This was particularly evident in the summer months. Children presenting for other reasons were also raising anxiety related issues with staff. Clinicians further recounted an increase in teenagers presenting with alcohol ingestion, incidents of self-harm, eating disorders and general anxiety:

*“I think the main group that has been struggling hugely here has been kind of teens really, early teens,”* [P11, Female MSW]

Cases of younger children presenting with anxiety and panic attacks was also highlighted:

*“9, 8-year-olds who shouldn’t really be anxious about things like this but come with severe anxiety and panic attacks*” [P6, Female EMC]

COVID-19 related anxiety, which in some instances exasperated existing conditions such as Obsessive-Compulsive Disorder (OCD), was also evident. Children held fears about the effect of COVID-19, voicing concerns about family members dying. A number of clinicians cited the closure of schools, social isolation and lack of routine as compounding children’s anxiety and psychological wellbeing, and the disruption of mental health services in the community leading to families presenting in crisis, having gone beyond their ability to cope.

### Disrupted access to community healthcare

#### Primary care

It became apparent to some clinicians that parents were having difficulty in accessing GP care, as parents reported being unable to secure an appointment, thus leaving them with little option but to attend the ED.

*“So, they [parents] would say like, ‘You know, I really didn’t want to come, I was trying to get a GP appointment but they’re maybe just doing virtual clinics or whatever, so they told me to come along”* [P11, Female MSW]

With regard to public health, difficulties in maintaining infant’s vaccination schedule was raised by one clinician who triaged patients, which parents explained as due to either an inability to secure an appointment with the GP or fear of attending the service. Those with new-born babies were also impacted by the suspension of public health nurse visits and some presented due to struggles with colic and feeding issues, compounded by not being able to rely on wider family or peer support networks:

*“These parents were first time parents, you know, they didn’t have the public health nurse they didn’t have the support, that they often came in and they were very, you could see they were very vulnerable*” [P8, Female NMS]

Restrictions on the delivery of primary and community health services left parents without what one clinician described as *“normal systems and supports”* [P13, Male EMC], and the effect of this was manifesting in the ED.

#### Loss of a safety net

Clinicians pointed to the closure of schools and suspension or restricted access to other community-based services, activities and supports, such as afterschool activities, and crucially, mental health services as impacting children’s health and wellbeing throughout the lockdown. One clinician explained how they treated a number of patients presenting with self-harm who were attending Child and Adolescent Mental Health Services (CAMHS) before the pandemic but were no longer attending:

*“It seemed like a lot of girls hadn’t been in contact with them since February, the CAMHS, the mental health services teams or they weren’t getting appointments. And they were falling through the net”* [P14, Female NS]

Another clinician expressed concern for children with neurodevelopmental disabilities such as autism, who were no longer attending specialist schools and other community supports:

*“so a few children with autism and because the specialist schools provide them with such a routine and provide the family with support there was a number of incidences of children and families actually coming in in crisis with behaviour that had escalated beyond the point they were able to control and beyond the point of them being able to cope”* [P13, Male EMC]

Clinicians also drew attention to children from more vulnerable backgrounds who they felt were at heightened risk during this period due to the lack of the safety net provided by schools and other community services by picking up on issues and linking families in with supports:

*“Some kids that we are getting because the school isn’t picking up on certain little things that should be addressed*” [P6, Female EMC]

One clinician felt some children had *“slipped through the net”* [P14, Female NS] due to being isolated, out of school and out of sight of teachers who would normally be in a position to recognise signs.

## Structural and process changes

### Re-configuring the ED to create capacity

#### Rapid and continual adaptation of protocols and pathways

*“In the space of about maybe four or five days, they had to completely re-jig the whole department, re-design the whole department, re-design rosters, re-design our own way of working”* [P9, Female EMC]

As evident in the above statement, the operational changes undertaken in EDs in March 2020 were significant and rapid. The implementation of new protocols and measures was portrayed as iterative, with one clinician explaining the process mapping was like “*shifting sands”*. As EDs act as the front door of hospitals, increasing isolation capacity was crucial to preventing COVID-19 transmission. Infrastructures were re-fashioned to facilitate this, with structures to separate patients constructed. All sites strengthened triage systems to stream suspected COVID--19 cases to zoned areas. This involved acquiring additional space within the hospital, splitting ED footprint over two or three different areas, creating staffing and resource implications. Challenges in managing different sites arose, with management staff describing how they had to ensure all areas were monitored, *“juggling”* staff to ensure safety and high-quality care delivery:

*“two different resuses [sic] in two different areas in the department, so that is a challenge for staff you know trying to get the skill mix to ensure you have staff looking after the patient who has resus experience and paediatric experience*” [P8, Female NMS]

#### Permission to innovate

The re-configuring of the ED both spatially and operationally was depicted in positive terms. Clinicians viewed the changes as innovative and adaptive, with teams pushed to think outside the box, invoking a sense of *“comradery*”, *“collaborative”* and *“adaptive”* leadership. Clinicians welcomed the additional isolation capacity and strengthened infection prevention and control measures. As funding was provided and greater support from management given, it was viewed as a window of opportunity to improve and enhance the infrastructural space:

*“people are really really trying to think of ways we can improve things which everyone always does but this is an actual, you know we are allowed to do this now”* [P5, Female NMS]

Nonetheless, difficulties were encountered, particularly early on due to changing criteria around personal protective equipment (PPE). This generated stress

among staff and posed a challenge for those in leadership roles tasked with communicating changes:

“*The messaging at the start was very mixed and I think the staff found that very frustrating from day to day.*” [P9, Female EMC]

The introduction of PPE and increased need for infection control changed how care was delivered. Wearing PPE had implications for clinical interaction, the donning and doffing of which is an arduous process that increased non-clinical time: “*it makes everything longer, and everything takes more time now”* [P15, Female NS].

In an effort to minimize wearing full PPE, reducing exposure and time spent with patients, clinicians clustered care delivery:

*“we are trying to put all our care into a little care package [] I’m clustering my care so I’m not going in four or five times”* [P2, Female NMS]

In one hospital, the Paediatric ED obtained a number of single occupancy rooms which provided isolation capacity in another section of the hospital. In order to facilitate clustered care, staff created *“grab bags”* that contained all necessary intervention procedural equipment, eliminating the need to collect items from different areas. Furthermore, rather than having a dedicated resuscitation area, they created a portable *“resus trolley”* which could be easily transported, reducing movement and potential virus transmission. Once again, this highlights the agility with which departments could reconfigure care delivery in response to the pandemic.

#### “More Isolation, More Cubicles, More Staff, More Space”: demands on resources

Clinicians felt the pandemic magnified the prevalent resource limitations they faced within the health system: *“It just laid bare the deficiencies and inefficiencies that were already there”* [P13, Male EMC]. The expanded footprint of the ED, new streaming processes, and higher infection control measures created greater demand on staffing levels, compounded by the need for staff to self-isolate if they displayed symptoms and being unable to attend work. While funding was provided for additional staff, recruitment was a *“struggle”,* leaving departments facing shortages of staff, and fears were expressed about the implications on patient safety. Even with the obtained space, capacity was reduced and with attendances increasing, the capacity to operate during winter was questioned:

*“We now only have a capacity for 31 patients in our department at any one time...[]..We looked at December last year..[] … 90 something per cent of the time there are more than 30 patients in the department … [] … So you can see where we are going to run into problems with that”* [P4, Female NMS]

Concerns about winter also extended to infection control, with clinicians indicating that EDs were struggling to implement social distancing, leaving some questioning the ability to maintain infection control standards:

*“My concern is we won’t have the staff to facilitate the standards we have at the moment with IP&C [Infection Prevention & Control] and social distancing and isolating patients, I think we are going to have a terrible winter*” [P12, Female MSW]

### Quality and safety in care delivery processes

#### Communication challenges

A number of challenges to the flow of care, particularly in relation to PPE, posed difficulty in communicating both with patients and colleagues. Clinicians explained the importance of engaging with younger children to gain trust, which was more difficult with PPE. This also extended to staff communication when treating patients, with clinicians stressing the importance of clear communication when administering drugs or other care. Many pointed out the inability to read facial expressions and other visual cues, thus the introduction of PPE demanded clearer communication mechanisms to prevent any risk to patients:

“*So now people are behind masks, they’re in robes, you can’t take it for granted what drugs or what they’re asking for, it could potentially lead to errors in care delivery because the communication now is different”* [P7, Female NMS]

In one hospital, communication between social workers, who are typically present in the ED, and clinicians was affected when social workers were relocated to another area, which was thought to impact the care flow of patients in need of social worker support. Concern was expressed that the extra steps required to refer patients to social workers no longer co-located may lead to delayed referrals.

The need to reduce footfall in EDs required staff to work in smaller teams in resuscitation scenarios, which was described by some as *“isolating*” and required strict management to ensure only necessary staff were present. One clinician highlighted the potential for errors with a reduced team:

*“We had a number of drug errors that probably shouldn’t have occurred cos normally in a resus scenario you would have, you know, people standing together double checking, cross checking but behind a door it’s very very difficult*” [P13, Male EMC]

### Psychological challenges for staff

For some clinicians, anxiety was a recurrent theme, with concerns voiced about contracting COVID-19 and transmitting it to their families or communities. Clinicians also had to adjust to reduced social interaction with colleagues, with break rooms no longer a place they could congregate. Clinicians highlighted that a strong team dynamic is essential when working in an ED, and peer support vital when dealing with adverse outcomes, thus reduced informal social interaction between colleagues was having an impact:

*“it was our room where we got to know our work colleagues you know …*. *or when we have a really traumatic event in resus and a patient dies it’s the place we used to go to kind of take a break and have a chat, a quick chat about it. That’s all gone, we can’t congregate at all”* [P4, Female NMS]

Uncertainty and anxiety were palpable for ED staff at the start of the pandemic, due to the media reports emerging from other European countries and the unknown of what was coming their way: *“we didn’t know what we were in for”* [P8, Female NMS]. As mentioned above, winter season brings extra challenges for staff every year, however this year will be even more challenging and concerns for staff wellbeing was voiced, as one clinician put it, as frontline staff, they questioned *“physically and emotionally what you can deliver”* [P5, Female NMS].

## Discussion

This qualitative study aimed to outline the experiences of frontline staff delivering care in order to gain further insight into the trends of paediatric presentations to the ED during COVID-19, and the work practice adaptations introduced in the ED in the response to the pandemic. The findings contextualise the reduced use of emergency care during the initial months of the pandemic, and provide evidence to suggest concerns regarding contracting COVID-19 and misinterpretation of public health messaging may have contributed to delayed presentations by paediatric presentations at the ED. A rise in attendances for mental health reasons was identified by clinicians, suggesting psychosocial implications for children. Disruptions to healthcare delivered in the community resulted in unmet healthcare needs for some children, and the suspension of schools and community support services removed a safety net for vulnerable children at risk of neglect. This study also identifies a number of structural and operational changes undertaken to increase isolation capacity in EDs, with streaming and zoning crucial to this process. This had staffing and spatial implications, and with attendances rising, concerns were raised about the ability of EDs to maintain current infection control standards, particularly during the winter season. A number of innovative initiatives to deliver care were highlighted, and quality and safety challenges around communication identified. Finally, working on the frontline in the midst of a pandemic created uncertainty and anxiety, significantly impacted staff wellbeing and rapport.

The cases of delayed presentations outlined by clinicians supports the findings of previous studies [5, 7, 8] and this study provides further insight into this phenomenon, highlighting the impact of parental hesitancy to attend the ED due to COVID-19 [9]. Public health messaging clearly impacted parents faced with making decisions around seeking care for children, thus clear and uncomplicated communication is essential [9]. The positive reaction to infection control measures displayed by parents suggests that a public communication strategy, clearly outlining the measures in place at the ED, can help to combat fears and reassure parents to continue to present [7]. While it is more difficult to adequately assess the extent of avoidance of emergency healthcare, it is vital that children with complex needs can continue to access appropriate healthcare. Future studies should investigate the impact of the pandemic on this particular cohort and their families.

Difficulty in accessing primary and community services, including GPs, public health nurses, mental health and other community health services may be further exasperating parents’ ability to access care for their children. Moreover, the reduction or suspension of educational, social and other community services and activities such as sports and youth outreach programmes, also raised issues around child protection for vulnerable children [8, 12], as illustrated by the findings of this study. The pandemic likely exacerbated existing economic and health inequities, further impacting marginalised children [20] and vulnerable children may have been at risk of *“slipping through the net”*. The impact of lockdown may also have been felt more keenly by children with disabilities and chronic health conditions, whose families rely on support services and specialist schools [13, 21]. It is imperative that both short- and long-term support measures and contingency plans are implemented to prevent and alleviate the negative effects on these children [22].

Widespread disruption to mental health services, including repurposing of staff and facilities, has been identified at a time when they may be needed more than ever [23]. The conditions of imposed “lockdowns” may be having a negative effect on children’s mental health [11], and as identified in this study and elsewhere [24], this is manifesting in rising psychosocial presentations at the ED, which may not be the most appropriate pathway to mental health care. As the pandemic continues to progress, policy makers and service providers must ensure the continued provision of essential health and social services, including targeted responses for those with existing conditions.

The implications of COVID-19 for PEDs were largely logistical. The scale of this pandemic has not been faced before in the western European context. A survey highlighted that planning and simulated responses were not carried out in over a third of PEDs across Europe, and guidelines that may be widely applicable across health systems to prepare and respond rapidly and effectively to the COVID-19 pandemic have been called for by those working in PEDs [14]. This study also highlighted the impact of resource poor services, particularly in relation to staffing levels and spatial limitations, which will progressively worsen during the winter season, bringing further logistical and clinical challenges [25], which must be considered in operational planning for further COVID-19 surges [26] and future public health crises. Strategies to lessen the burden on EDs through public health measures, strengthened access to primary care services, and other ways to deliver care must be enacted if children’s healthcare needs are going to be sufficiently met. Finally, those working in paediatric EDs have been somewhat overlooked due to the lesser clinical impact of COVID-19 on children, and this study also provides evidence to suggest the impact on staff is considerable and should be examined further.

### Strengths and limitations

This study provides rich insight from frontline staff working in Ireland, however as the results are consistent with previous studies carried out in other countries [7, 8, 14] they may be applicable in other contexts. The multidisciplinary nature of the clinicians included in this study reflects a wide range of experiences. The findings are based on frontline staff’s perception of parents’ health seeking behaviour, and future research could further understand in this area. Data collection occurred at one point of time, after the first and most challenging wave when health services were largely unprepared for the pandemic.

## Conclusion

The spread of COVID-19 and subsequent polices to address the pandemic has had wide-reaching implications for children’s health and wellbeing. Public health messaging has a role to play in ensuring children continue to receive appropriate healthcare when restrictions are placed on movement. The interruption to healthcare delivered in the community is manifesting in myriad ways in the ED, including in the rise of psychosocial presentations in children. EDs have shown innovation and agility in the structural and operational changes they have implemented to continue to deliver care to children, however resource limitations and other challenges must be addressed to ensure high quality care delivery and protect the wellbeing of those tasked with delivering this care.

## Supplementary Information

**Additional file 1.**

## Data Availability

The datasets used and/or analysed during the current study available from the corresponding author on reasonable request.

## Abbreviations

ED: Emergency department
GP: General practitioner
ICU: Intensive care unit
PED: Paediatric emergency department
PPE: Personal protection equipment
