# Supplementary material for: The impact of the COVID-19 pandemic on child health and the provision of Care in Paediatric Emergency Departments: a qualitative study of frontline emergency care staff
Source: BMC Health Serv Res. 2021 Mar 25;21:279. doi: 10.1186/s12913-021-06284-9 (PMC7993902; doi:10.1186/s12913-021-06284-9)
Supplement: Supplementary file 1 — Additional file 1. [file 12913_2021_6284_MOESM1_ESM.docx]

**Supplementary Material: Interview Topic Guide**

1. Can you talk about your experience of ED attendance throughout the Covid-19 pandemic?
   1. Can you tell me about changes in attendance patterns throughout the implementation of various public health stages in response to the COVID-19 pandemic?
   2. Are there any particular characteristics of these changes, for example patient groups or presenting conditions?
   3. Has there been any other changes to patient’s behaviour throughout the pandemic?
   4. Have you noticed any change in parents’ behaviour when attending the ED?
   5. Have parents expressed any concerns about using the ED during this time?
2. Do you think there are any patient groups at risk of avoiding the ED at this time?
   1. If so, who are they and why are they at risk?
   2. How do you think these patients could be supported during this time?
3. Can you talk about the measures or operational changes that have been implemented in your workplace regarding the management of paediatric patients since the onset of COVID-19?
4. How has the way you work changed?
5. How have you found working under these/any changes?
6. How have the changes impacted paediatric patients?
7. What have been the advantages of the implemented changes?
8. Has there been any disadvantages in relation to the changes?
9. Is there anything you think should have been done differently?
10. Are there any operational changes that you would like to see retained on a permanent basis? If so, why?
11. Do you have anything else to add regarding paediatric patients presentations to the ED during COVID-19?
